# Integration of morphologic and genetic data clarifies the evolution and species boundaries within a *Nychiodes* Lederer, 1853 species complex (Lepidoptera, Geometridae)

**DOI:** 10.3897/zookeys.1273.161858

**Published:** 2026-03-17

**Authors:** Dominic Wanke, Simon Müller, Sajad Noori, Ricardo J. Pereira, Hossein Rajaei, Pasi Sihvonen, Kyung Min Lee

**Affiliations:** 1 State Museum of Natural History Stuttgart, Rosenstein 1, D-70191 Stuttgart, Germany University of Hohenheim Stuttgart Germany; 2 Center of Excellence for Biodiversity and Integrative Taxonomy (KomBioTa), Wollgrasweg 23, D-70599, Stuttgart, Germany University of Helsinki Helsinki Finland; 3 University of Hohenheim, Garbenstraße 30, 70599 Stuttgart, Germany State Museum of Natural History Stuttgart Stuttgart Germany; 4 Finnish Museum of Natural History, University of Helsinki, P.O. Box 17, FI-00014, Helsinki, Finland Center of Excellence for Biodiversity and Integrative Taxonomy (KomBioTa) Stuttgart Germany

**Keywords:** DNA barcoding, ecological niche modeling, integrative taxonomy, multispecies coalescent analysis, phylogenetic analysis, species delimitation

## Abstract

Young species radiations are valuable from an evolutionary perspective, as they can reveal traits involved in species formation, but they are challenging for taxonomists due to discordance among independent lines of evidence. The present study integrates morphologic, ecological, and phylogenetic data to resolve delimitation of species within the geometrid moth genus *Nychiodes* Lederer, 1853, with a particular focus on the closely related species pair *N. divergaria* Staudinger, 1892 and *N. subvirida* Brandt, 1938. Our findings revealed significant ecological niche differentiation across the Iranian mountain ranges between the range of taxa distinguished by morphology and DNA barcodes. While individual gene trees from mtCOI and nuclear loci showed conflicting results regarding species boundaries, our multispecies coalescent analyses support the distinction of taxa with different female genitalia characters. Our study highlights how the combination of morphological, genetic, and ecological data helps to define the boundaries between closely related species, to solve taxonomic uncertainties in regions with a complex evolutionary background.

## Introduction

The use of morphological characters has traditionally been a cornerstone in taxonomy for the identification and delimitation of insect species (e.g. Cook et al. 2010). Initially, taxonomists relied on external morphological characters, which provided sound criteria for delimiting well-divergent insect taxa. However, these traits were often insufficient for differentiating closely related species or genera until genitalia structures were incorporated, revealing additional crucial diagnostic characters (e.g. Pierce 1914; Hardwick 1950; Sihvonen 2001; Wipfler et al. 2016). While such internal genitalia characters can reveal how selection promotes species formation, e.g., through sexual selection, their interpretation can be complicated by recent divergence times between sister species, evolutionary constraints due to their core role in reproduction, or because of selection in such traits that may distort the inferred evolutionary history of the diverging species (Smith et al. 2011; Hench et al. 2022). Integrative taxonomy, which consider different taxonomic criteria, such as internal morphological characters, independent genetic markers, and ecological features of their distribution, is an important tool to overcome these challenges towards an understanding of species boundaries and their evolution.

Among these, mitochondrial DNA barcoding of COI has been particularly useful as a rapid and cost-efficient molecular tool for species identification (Rajaei 2015; Antil et al. 2023; Yang et al. 2024). Moreover, these barcodes are collected and curated in online libraries (such as BOLD, https://boldsystems.org/), serving as reference databases that assist with species discovery and monitoring (Antil et al. 2023; Yang et al. 2024). Because barcode genes, such as COI, tend to exhibit higher divergence between species than within species, they provide a rapid and reliable approach for species identification, even in cases where morphological differences are subtle, while acknowledging that some intraspecific variation may be present (Hebert et al. 2003a; Kress and Erickson 2008). Nevertheless, DNA barcoding has inherent limitations. Its effectiveness depends on a comprehensive reference database, which often lacks entries for rarely collected or newly described species (Hebert et al. 2003b). Moreover, several genetic and evolutionary factors, such as pseudogenes, intraspecific variation, and introgressive hybridization, complicate species delimitation by causing phylogenetic mismatches between morphological and molecular data (Maddison 1997; Meyer and Paulay 2005; Rubinoff et al. 2006; Song et al. 2008; Degnan and Rosenberg 2009; Yang et al. 2024). These challenges are particularly significant in groups characterized by large population sizes and recent diversification events, where incomplete lineage sorting (ILS), and therefore discordance among independent gene trees, is more common. In Lepidoptera taxonomy, COI based DNA barcoding is widely used across families (e.g. Hausmann et al. 2011; Lukhtanov et al. 2025), although it may be less effective in cases of recent speciation or where multilocus markers better reflect species boundaries.

**Figure 1.**
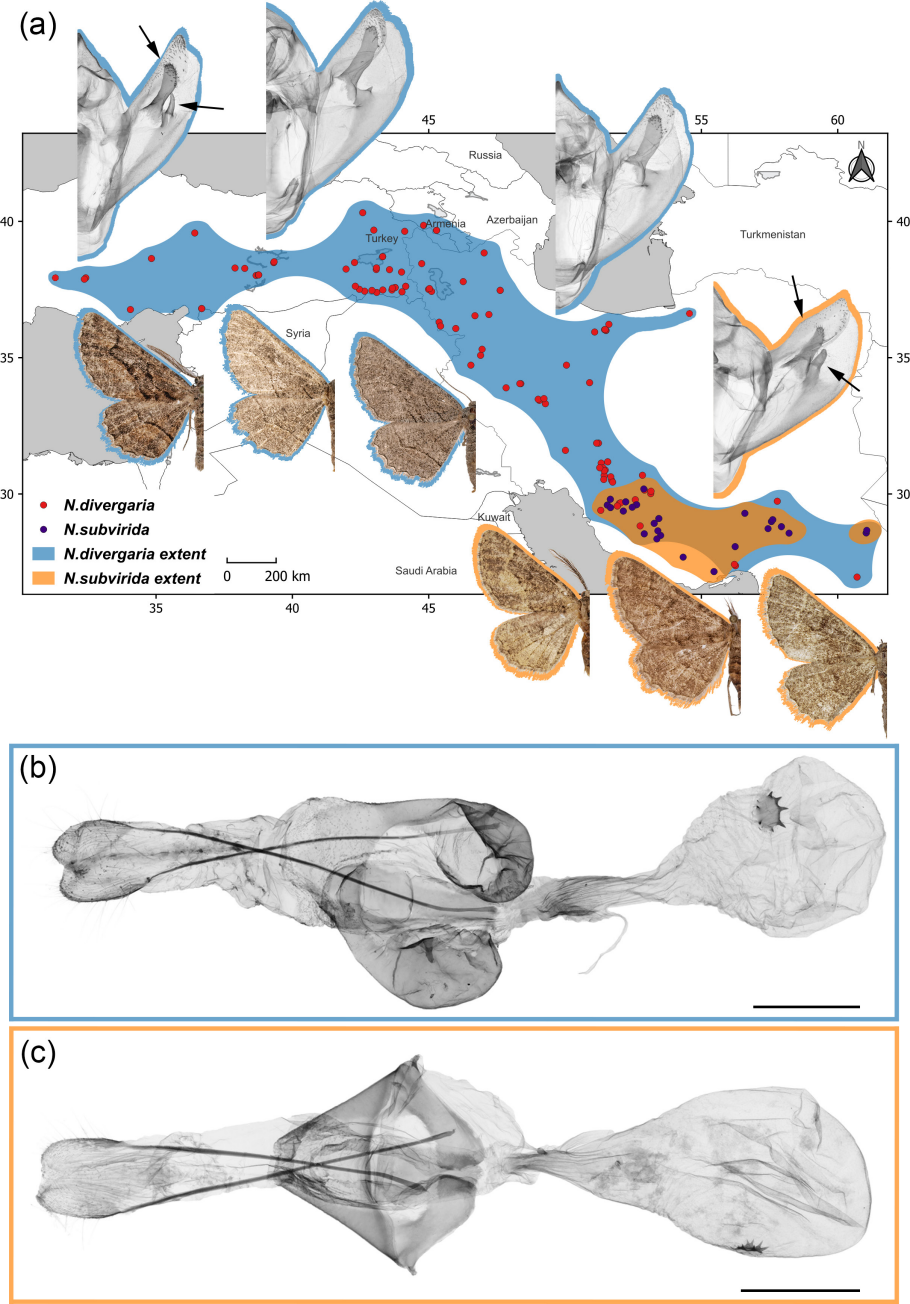
Distribution ranges and morphological characters of the studied *Nychiodes* species, *N. divergaria* and *N. subvirida*. **a**. Examined records and extrapolated distribution ranges. Wing pattern and the male genitalia capsule are marked with blue outlines for *N. divergaria* and with orange outlines for *N. subvirida*. Arrows on the male genitalia capsule indicate structural variability in details of valva. Distribution ranges were generated by a buffer of 50 km around examined records; **b**. Female genitalia of *N. divergaria*; **c**. Female genitalia of *N. subvirida*. Wing and male genitalia photographs are not to the same scale. Scale bars: 1 mm (**b, c**).

Recently, it has been shown that the geometrid moth genus *Nychiodes* Lederer, 1853 contains species with high intraspecific variation in external and internal genitalia characters, which hamper their identification (Wanke et al. 2020). This genus consists of 25 species distributed from Western Europe and North Africa to Iran, Afghanistan and Pakistan (Müller et al. 2019; Wanke et al. 2020; Rajaei et al. 2023). Within this genus, a comprehensive examination of numerous specimens, including their genitalia, has shown that most species occur in the Middle East (Wanke et al. 2020). Larvae of *Nychiodes* species are characterized by an orange collar, while their adults are distinguished by prominent ante- and postmedial lines on the forewings (Müller et al. 2019; Wanke et al. 2021). *Nychiodes divergaria* Staudinger, 1892, shows significant intraspecific variation in both wing pattern and male genitalia morphology, challenging its delimitation where it is sympatric with its sister species. This species is distributed from southern Turkey and southern Armenia along the Zagros Mountains in Iran to the border of Pakistan and along the Alborz mountains in northern Iran (Wanke et al. 2020; Fig. 1). In southern Iran, *N. divergaria* is sympatric with its sister species, the endemic *N. subvirida* Brandt, 1938 (Wanke et al. 2020; Fig. 1). However, the female genitalia show clear diagnostic characters for species delimitation, with two sclerotized spherical patches in *N. divergaria* that are not present in *N. subvirida* (Fig. 1), whereas COI barcodes are intermixed, suggesting either recent speciation with ILS or mitochondrial introgression across species boundaries (Wanke et al. 2020).

To robustly delimit these taxonomically challenging species, we employed complementary approaches. First, we analyzed distribution data to test the null hypothesis that the putative species share the same ecological niche, with the alternative hypothesis that niche divergence facilitated species formation. Second, we used a multispecies coalescent model of independent nuclear genes to test the monophyly of the putative species. Together, these approaches evaluate whether morphological and genetic polymorphism reflects recent speciation events.

## Material and methods

### Occurrence data

We compiled the current distribution of *Nychiodes divergaria* and *N. subvirida* across their respective ranges. Our dataset includes 126 occurrences for *N. divergaria* and 31 for *N. subvirida*, identified using morphology and DNA barcodes from the study by Wanke et al. (2020), which are in strong concordance. After removing duplicated and missing values for longitude and latitude, we finalized the dataset with 110 occurrences for *N. divergaria* and 27 for *N. subvirida*.

### Variable selection

We tested 19 high-resolution bioclimatic variables from the Chelsa dataset (approximately 1 × 1 km^2^; Karger et al. 2017) to identify variables with higher contribution and minimal multicollinearity issues using various analyses. First, using pairwise correlation tests (*r* > 0.75), we investigated the correlation among different variables using cor function from corrplate package (v. 0.92; Wei and Simko 2021). Then we shortlisted the variables with minimum collinearity issues using the variation inflation factor (VIF) from usdm package (Naimi et al. 2014). Finally, we checked the contribution of each variable across the climate space by principle component analysis (PCA) using dudi.pca function from ade4 R package (Dray and Dufour 2007; Noori et al. 2024a, 2025; R Core Team 2024; Suppl. material 1: fig. S1). We selected seven variables for *N. divergaria* and six for *N. subvirida* (Table 1; for more details see the Suppl. material 1: fig. S1).

**Table 1.** List of selected variables for the studied species of *Nychiodes*.

Species	Selected variables
*N. divergaria*	Bio3: Isothermality
	Bio7: Temperature annual range
	Bio8: Mean temperature of wettest quarter
	Bio9: Mean temperature of driest quarter
	Bio12: Annual precipitation
	Bio14: Precipitation of driest month
	Bio15: Precipitation seasonality (coefficient of variation)
*N. subvirida*	Bio3: Isothermality
	Bio7: Temperature annual range
	Bio9: Mean temperature of driest quarter
	Bio14: Precipitation of driest month
	Bio15: Precipitation seasonality (coefficient of variation)
	Bio16: Precipitation of wettest quarter

### Species distribution models (SDMs)

Using occurrence data and selected environmental covariets, we modeled the current habitat suitability for the studied species. Following previous studies (Ginal et al. 2022; Noori et al. 2024a, 2024b, 2025), we modeled species distributions using the maximum-entropy algorithm (MaxEnt) implemented through the biomod2 package in R (R Core Team 2024; Thuiller et al. 2024). MaxEnt has been widely used for modeling species habitat suitability, particualry in sthe case of presence-only data (Phillips et al. 2009; Fourcade et al. 2014; Noori et al. 2024b; Yang et al. 2024).

However, in our analysis, we implemented several strategies to reduce the uncertainty and potential biases (see SM for more details). First, 10,000 background/pseudo-absence points were randomly sampled across the accessible area for the studied species. Next, we constructed ensemble final models by selecting models with higher performance across 100 replicates, as outlined by Noori et al. (2024b, 2025). To do so, we only used the model with higher values of area under the ROC Curve (AUC_test_ > 0.7). The resulting habitat suitability maps were subsequently thresholded by applying a 10% omission rate. Finally, using a multivariate environmental similarity surface (MESS) from dismo package, we defined the areas within and outside of the training range (Suppl. material 1: figs S2, S3; Elith et al. 2010; Rödder et al. 2013; Ginal et al. 2022).

### Niche and hypervolume assessment

**Figure 2.**
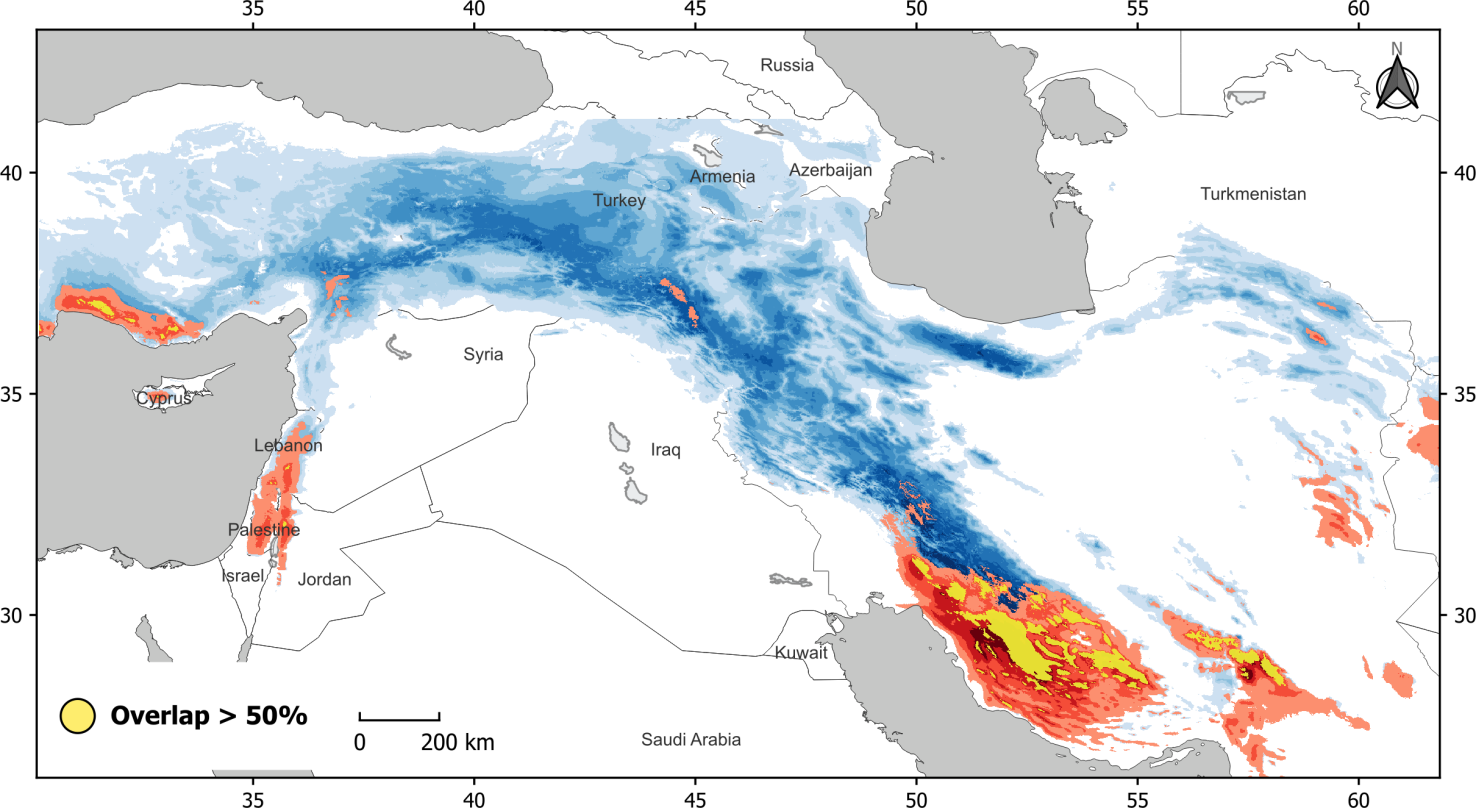
A map for habitat suitability of *N. divergaria* (blue) and *N. subvirida* (red). The white area show the unsuitable habitats for both species. Yellow color indicates overlapped areas with higher habitat suitability (>50%) between two species.

The occupied climate space and hypervolume of the *Nychiodes* species were assessed to define the similarity/dissimilarity volume and overlaps of their niches based on SDM results (Fig. 2, Table 3). The resulting SDMs habitat suitability of the species were masked by a Minimum Convex Polygon (MCP) based on a buffer of 50 km around the species occurrences using rangeBuilder package (v. 2.1; Davis Rabosky et al. 2016). Using two thresholds of 0.25 and 0.5, a binary presence-absence raster was generated. Next, 1,000 coordinated points were randomly selected across the species range. The climate values of these points were extracted from the shared bioclimatic variables between species (bio3, 7, 9, 14, and 15). This dataset was used to reduce the multidimention of the climate space using PCA for further steps (detailed in SM).

**Table 2.** The average values for TSS and AUC, and contribution of selected environmental variables, for *Nychiodes* species. The contributions of dominant climate variables to the model are displayed in bold.

Species	TSS	AUC	bio3	bio7	bio8	bio9	bio12	bio14	bio15	bio16
*N. divergaria*	0.67	0.91	6.70	20.1	0.159	8.28	23.4	3.65	11.0	—
*N. subvirida*	0.81	0.97	0.06	16.1	—	0.56	—	7.93	31.4	17.9

**Table 3.** Results of Wilcoxon test and Schoener’s *D* and Hellinger’s *I* using the first two PCA components for habitat (dis)similarity and the occupied hypervolume for each species and their similarity and intersection of *Nychiodes divergaria* and *N. subvirida*.

Threshold	*p*-value	*D*	*I*	*N. divergaria* volume	*N. subvirida* volume	Jaccard	Sørensen	Intersection
>50%	<0.001***	0.38	0.61	28.71	12.44	0.33	0.49	10.12
>25%	<0.001***	0.33	0.57	30.02	10.31	0.30	0.47	9.39

Niches of both species were created using ecospat.grid.clim.dyn function of ecospat package based on the first two PCA components (v. 4.1.1; Di Cola et al. 2017; Broennimann et al. 2024). Niche overlaps were assessed using two indices for similarity (Hellinger’s *I* (*I*)) and dissimilarity (Schoener’s *D* (*D*)) of species niches using ecospat.niche.overlap function (Broennimann et al. 2024). These metrics indicate the similarity (*I*) and dissimilarity (*D*) of niches between species (Di Cola et al. 2017; Table 3). Then, we performed a non-parametric Wilcoxon test to determine if species niches between the two species significantly differ (Patil 2021).

Finally, we quantified the occupied climatic hypervolume of each species and their degree of (dis)similarity using the first two principal component (PC) axes. Species hypervolumes were estimated using the hypervolume_gaussian function from the hypervolume package (v. 3.1.6; Blonder et al. 2025), based on 1,000 stochastic samples per species. We then quantified hypervolume size, intersection, and similarity between species using Jaccard and Sørensen indices at both suitability thresholds (Blonder et al. 2025; Table 3).

### Molecular data generation

DNA extraction and amplification of the barcode fragment (658 base pairs of the 5' terminus) of the mitochondrial cytochrome c oxidase subunit I (COI) were conducted following the manufacture’s protocol of the DNeasy Blood and Tissue kits (Qiagen, Hilden, Germany) using a leg from a single dry collection specimen in the framework of the revision of the genus *Nychiodes* (Wanke et al. 2020). Amplification of nuclear markers was conducted following Wahlberg and Wheat (2008) and Wahlberg et al. (2016) using the above-mentioned extracts. We amplified and sequenced five protein-coding nuclear gene regions for downstream analyses: arginine kinase (ArgK), sarco/endoplasmic reticulum calcium ATPase (Ca-ATPase), elongation factor 1 alpha (EF-1alpha), ribosomal protein (RpS5) and wingless (wgl) for downstream analyses. Sequence alignments for each gene were performed using the Muscle algorithms as implemented in MEGA11 (Tamura et al. 2021). Four sister species (*Nychiodes leviata* Brandt, 1938, *N. farinosa* Brandt, 1938, *N. antiquaria* Staudinger, 1892, and *N. admirabila* Brandt, 1938) were used as outgroup for phylogenetic analysis. GenBank accession numbers for sequences used are presented in Suppl. material 1: table SS2.

### Phylogenetic analyses

For the maximum-likelihood (ML) analysis, the six concatenated gene sequences were analyzed using IQ-TREE v. 2.1.3 (Minh et al. 2020). The best partitioning scheme for each gene’s codon position was identified with ModelFinder (Kalyaanamoorthy et al. 2017) and selected based on the corrected Akaike Information Criteria. We used the ultrafast bootstrap approximation method (UFBoot2) to assess branch support with 1,000 replicates (Minh et al. 2013). The best-fitting substitution models for each gene were as follows: COI, TPM2u+F+I; ArgK, HKY+F+I; Ca-ATPase, JC; EF-1alpha, TNe+I; RpS5, K2P+I; wgl, TN+F+I. The resulting trees were visualized and rooted to the outgroups mentioned above using FigTree v. 1.4.3 (Rambaut 2021) and further modified in CorelDRAW v. 24.

In addition, to account for possible incomplete lineage sorting common in recent speciation events, a coalescent-based method was employed to estimate the species tree. Gene trees for four nuclear genes, excluding ArgK and Ca-ATPase genes due to large data omissions, were generated via IQ-TREE with 1,000 ultrafast bootstraps and ModelFinder. ASTRAL-III v. 5.7.8 (Zhang et al. 2018) was used to infer species trees for both data before and after the removal of recombinant loci from multi-individual dataset (Rabiee et al. 2019) with 100 replicates of multi-locus bootstrapping (Seo 2008).

### Species delimitation (SD) analyses

We conducted the analyses using three complementary approaches: a distance-based method (ABGD) based on mtCOI and two multispecies coalescent methods (bPTP and GMYC) that incorporated multi-locus data, combining mtCOI with five nuclear gene data. Automatic barcode gap discovery (ABGD) analysis was performed under the K2P model. The parameter settings were as follows: *P*_min_ = 0.001, *P*_max_ = 0.1, steps = 10, relative gap width = 1.5, number of bins = 20, with all other settings at default. Both bPTP (Bayesian Poisson Tree Processes) and GMYC (Generalized Mixed Yule-Coalescent) require an ultrametric tree. To obtain this, we used the ML tree generated by IQ-TREE, and subsequently applied a molecular clock transformation via ‘chronos’ function in the R package ape (Paradis et al. 2004). The bPTP analysis was conducted using the online web server (https://species.h-its.org/) with the ultrametric tree as input. The Markov chain Monte Carlo (MCMC) chain was run for 200,000 generations, with a thinning interval of 1000, a burn-in of 20%, and a default random seed. Outgroup taxa were removed prior to analysis. The GMYC analysis was performed using the single-threshold model implemented in the R package splits (Ezard et al. 2009), with the ultrametric tree as input after outgroup removal.

### Species network analysis using mtCOI

Relationships between haplotypes of *Nychiodes divergaria* and *N. subvirida* were assessed using a median-joining haplotype network created with PopArt v. 1.7.2 (Leigh and Bryant 2015) based on mtCOI sequences.

## Results

### Niche differentiation

Through our variable selection, distinct sets of climate variables were selected for each studied species of *Nychiodes* (Table 1). Five variables, which made significant contributions, are shared between the two species, while additionally variables are specific to each (Suppl. material 1: table SS1, fig. S2), consistent with niche divergence between taxa. The contribution values of the climate variables in SDMs shows that the variables Bio7 (Temperature Annual Range) and Bio12 (Annual Precipitation) have the highest contribution for modeling *N. divergaria*, whereas Bio15 (Precipitation Seasonality) and Bio16 (Precipitation of Wettest Quarter) are most influential for *N. subvirida* (Table 2). Except for Bio3 and Bio9, the other shared variables between the two species with highest contribution are completely opposing each other. For example, Bio12, which has the highest contribution to models of *N. divergaria*, are exactly in opposition to this variable in *N. subvirida* (Suppl. material 1: fig. S1b, d). Similar patterns can be seen for Bio7 and Bio16. Overall, our findings indicate that *N. divergaria* occupies wetter niches across a broader climate niche space, while *N. subvirida* extends across drier and narrower niches.

The resulting ensemble models indicate differing habitat suitability across the distribution ranges and in the overlapping areas of the two species (Fig. 2, Suppl. material 1: figs S2, S3). For *N. divergaria*, regions of higher habitat suitability extend from southern Turkey, through the central Zagros and Alborz Mountain ranges. In contrast, *N. subvirida* is mainly restricted to the southern Zagros Mountain and some mountains in southeastern Iran (Suppl. material 1: figs S2, S3). Overall, in the overlapping areas (Fig. 2), *N. divergaria* predominantly occupied the eastern slopes of the Zagros Mountains, while *N. subvirida* is primarily distributed along the southern slopes of the Zagros Mountains, extending towards coastal areas of the Persian Gulf.

### Niche overlap and hypervolume

As shown in Table 3, the niches (occupied climate spaces) of two species are significantly different at two habitat suitability thresholds, supporting the niche divergence hypothesis. The two species partially overlap in their climate spaces across Zagros Mountain (Fig. 2), where *N. divergaria* occupies a broader climate niche space compared with *N. subvirida* (see Suppl. material 1: figs S5–S12, for more details).

### Genetic data

**Figure 3.**
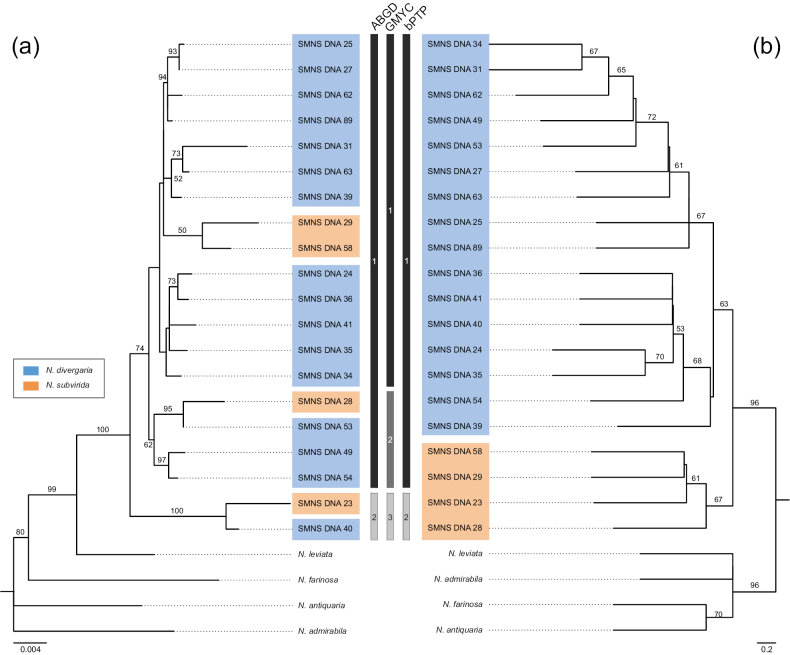
Phylogenetic relationships and species delimitation (SD) within the *Nychiodes divergaria–subvirida* complex based on a multi-individual dataset. **a**. Maximum-likelihood (ML) tree inferred with IQ-TREE from a concatenated alignment including COI and five nuclear gene sequences; **b**. Species tree inferred under the multispecies coalescent model using ASTRAL, based exclusively on genes from four nuclear loci (ArgK and Ca-ATPase excluded due to missing data). The middle panel presents SD analyses based on ABGD (COI only) and GMYC and bPTP (multilocus dataset). Species delimitation bars correspond to the individuals shown in the ML tree (left panel). Each bar reflects the number of delimited species (indicated by numbers) and the corresponding grouping of individuals.

The ML tree based on the six concatenated gene dataset (mitochondrial COI and nuclear five genes) supports the *N. divergaria–subvirida* complex as a monophyletic group with 100% bootstrap support (Fig. 3a). Within this lineage, the two species are intermixed, likely reflecting discordance between mitochondrial and nuclear signals in the concatenated analysis. In contrast, the species tree constructed using ASTRAL based exclusively on four nuclear loci clearly separates the two species (Fig. 3b), though with moderate bootstrap support (63% for *N. divergaria* and 67% for *N. subvirida*). The *N. divergaria*–subvirida lineage remains strongly supported in the multispecies coalescent tree inferred with ASTRAL, with 96% bootstrap support. The four sister species—*N. leviata*, *N. farinosa*, *N. antiquaria*, and *N. admirabila*—are clearly separated from the *N. divergaria–subvirida* lineage and served as outgroups in both the ML and coalescent-based analyses.

The current classification of the *Nychiodes divergaria–subvirida* complex, based on morphological traits, was not supported by molecular species delimitation (SD) analyses. Instead, SD analyses suggested completely different groupings of individuals. Both ABGD (based on mitochondrial COI) and bPTP (based on multigene data) identify the same two groups with identical individual compositions, whereas GMYC (also based on multigene data) further subdivided one of these groups (Fig. 3, middle bars).

*N. divergaria* and *N. subvirida* share COI haplotypes and show high external morphological variation, but male genitalia and nuclear DNA support their distinction. Ecologically, divergaria mostly overlaps with subvirida, indicating limited niche divergence.

## Discussion

The present study highlights the importance of integrative taxonomic approaches to understand species differentiation in recent speciation events. *Nychiodes divergaria* and *N. subvirida* share COI haplotypes and show high external and internal (male genitalia) morphological variation, but female genitalia (see Wanke et al. 2020) and nuclear DNA support their distinction. Ecologically, *N. divergaria* and *N. subvirida* show a considerable geographic overlap, particularly at higher elevations, indicating that spatial co-occurrence alone does not necessarily reflect ecological similarity. COI discordance likely reflects incomplete lineage sorting or recent introgression, which is expected in recently diverged species pairs.

The niche qualification test revealed significant ecological niche divergence between the two species (Table 3). While temperature- and precipitation-related variables jointly influence the distribution of *N. divergaria*, the distribution of *N. subvirida* is mainly determined by precipitation-related variables (Table 2). In addition, opposite directions of PCA loadings of climate variables indicate that the species occupy distinct climate niche spaces (Suppl. material 1: figs S2, S3), despite their partial geographic overlap. A possible interpretation would be that *N. divergaria* is associated with relatively wetter climatic conditions, whereas *N. subvirida* tends to occur in comparatively drier environments (Suppl. material 1: fig. S1).

This ecological differentiation is likely linked to habitat and bioclimatic region differentiation across the Zagros Mountains, a major biogeographic transition zone in this region. The southern Zagros, with a hotter and drier climate, is located at a transition zone between Mediterranean and subtropical macroclimate regions along the northern costal of the Persian Gulf. On the other hand, northwestern slopes of Zagros Mountain have more Mediterranean climate condition with higher precipitation (Djamali et al. 2011). Furthermore, the overlapping areas between the two species extend across two different ecoregions: Zagros Mountains Forest steppe and Nubo-Sindian desert and semi-deserts (Fig. 2; Dinerstein et al. 2017; Noori et al. 2025). Accordingly, although the two species may co-occur at similar elevations, they occupy different ecological niches, with *N. divergaria* predominantly associated with the eastern slopes of the Zagros Mountains and *N. subvirida* occurring mainly on the western and southern slopes of the mountain ranges. Previous studies have shown that Zagros Mountains serves as a biogeographic barrier and as a corridor at the intersection of two biogeographical realms, the Palearctic and the Saharo-Arabian, which has dramatically shaped the biodiversity patterns in western Asia (Noori et al. 2024c, 2025). The observed niche differentiation between *N. divergaria* and *N. subvirida* likely reflects this complex role of the Zagros Mountains as a dynamic transition zone rather than a strict dispersal barrier. The constructed species tree, based on four nuclear loci, clearly separates *N. divergaria* and *N. subvirida*, albeit with a moderate bootstrap support. Species delimitation remains challenging in recently diverged taxa due to discrepancies among different datasets and analytical methods.

While traditional taxonomy based on external and genitalia morphology (Wanke et al. 2020) and analyses of nuclear markers confirm two distinct lineages, COI data show discordance, which more likely reflect mitochondrial introgression, and are therefore misleading in delimiting species.

Notably, characters of the female genitalia aligned well with the nuclear species tree inferred under the multispecies coalescent framework, that incorporates discordance among gene trees caused by likely introgression. The finding suggests that female genitalia represent a relatively conserved and reliable morphological trait for species delimitation in this group. The sharing of mitochondrial lineages between *N. divergaria* and *N. subvirida* likely reflects mitochondrial introgression, rather than evidence against species-level separation.

However, multispecies coalescent method, such as GMYC, should be interpreted with caution, as they have a known tendency to oversplit species (Dellicour and Flot 2018). Similar patterns have been reported in other insect groups, such as recently diverged grasshopper lineages (Nolen et al. 2020), indicating that this issue extends beyond our focal group and represents a broader challenge in species delimitation across insects. These inconsistencies highlight the limitations of relying solely on mitochondrial or limited nuclear data and emphasize the importance of integrating multiple lines of evidence, including morphology, distribution, ecology, and broader genomic data, to achieve robust species boundaries. Given the observed conflicts, which are relatively common in many insect taxa, future studies incorporating additional nuclear markers or genome-wide data may provide further insights into species limits and the complex evolutionary dynamics of this group. Considering all available data, including areas of conflict among datasets, we follow the principles of integrative taxonomy (Padial et al. 2010) and regard *N. divergaria* and *N. subvirida* as two separate species. Here we point out the challenges inherent in species delimitation in a case of recent divergence and different type of data allow for different interpretations. Despite the significant intraspecific morphological variation, female genitalia remain a reliable diagnostic trait for defining species boundaries in this genus (see Wanke et al. 2020). Furthermore, although the two species partially share their geographic range, they occupy distinct ecological niches shaped by climatic differentiation across the Zagros Mountains. The transitional zone between Mediterranean and tropical macroclimate appears to have played a crucial role in driving ecological divergence, thereby contributing to the evolutionary trajectories and todays distributions of the two species (Noori et al. 2024c). This study provides further evidence that incongruence among datasets is an expected outcome in young species pairs and demonstrates that multispecies coalescent approaches, when interpreted in an integrative framework, are valuable tools for clarifying species boundaries in emerging species.

## Additional information

### Use of AI

No use of AI was reported.

### Author ORCIDs

Dominic Wanke https://orcid.org/0000-0001-5390-8993

Simon Müller https://orcid.org/0009-0009-9474-5516

Sajad Noori https://orcid.org/0000-0002-9933-6713

Ricardo J. Pereira https://orcid.org/0000-0002-8076-4822

Hossein Rajaei https://orcid.org/0000-0002-3940-3734

Pasi Sihvonen https://orcid.org/0000-0003-2237-9325

Kyung Min Lee https://orcid.org/0000-0001-9055-1073

### Data availability

All of the data that support the findings of this study are available in the main text or Supplementary Information.

## Supplementary materials

Supplementary material 1 Additional analyses Data type docx Explanation note Supplementary material to Integration of morphologic and genetic data clarifies the evolution and species boundaries in Nychiodes Lederer, 1853 (Lepidoptera: Geometridae). https://binary.pensoft.net/file/1563560This dataset is made available under the Open Database License (http://opendatacommons.org/licenses/odbl/1.0/). The Open Database License (ODbL) is a license agreement intended to allow users to freely share, modify, and use this Dataset while maintaining this same freedom for others, provided that the original source and author(s) are credited. Dominic Wanke, Simon Müller, Sajad Noori, Ricardo J. Pereira, Hossein Rajaei, Pasi Sihvonen, Kyung Min Lee
