# Supplementary material for: Integration of morphologic and genetic data clarifies the evolution and species boundaries within a Nychiodes Lederer, 1853 species complex (Lepidoptera, Geometridae)
Source: Zookeys. 2026 Mar 17;1273:167–83. doi: 10.3897/zookeys.1273.161858 (PMC13014137; doi:10.3897/zookeys.1273.161858)
Supplement: Supplementary material 1 — Additional analyses [file zookeys-1273-167_article-161858__-s001.docx]

# Supplementary Material 1

## Variable selections

We used the high resolution 19 bioclimatic variables from Chelsa dataset (Karger et al. 2017; for more details, see https://chelsa-climate.org). The climate data were generated for the period of 1981-2010, which overlap with the sampling period of *Nychiodes* species. The resolution of all utilized raster files was at 30 arc sec (WGS84; 1 x 1 km^2^). We used different methods to select the most independent environmental variables for each studied species. Using Pairwise correlation test by *cor* function from *corrplot* package, a correlation plot was generated to check the multicollinearity in the selected variables (Fig. b & d). In the next step, we checked the magnitude and direction of this collinearity using the Principal Component Analysis (PCA) embedded in *dudi.pca* function from *ade4* R package (R Core Team 2024; Dray & Dufour, 2007; Fig. S1). Finally, we shortlisted the variables with the least multicollinearity problems (less than 5) using Variation Inflation Factor (VIF) test in *usdm* package (Table S1).

**Table S1.** List of the selected environmental variables using Variation Inflation Factor Test (VIF).

| **Species** | **Selected variables** | **VIF** |
| --- | --- | --- |
| *N. divergaria* | Bio3: Isothermality  Bio7: Temperature annual range  Bio8: Mean temperature of wettest quarter  Bio9: Mean temperature of driest quarter  Bio12: Annual precipitation  Bio14: Precipitation of driest month  Bio15: Precipitation seasonality (coefficient of variation) | 2.00  1.55  2.91  2.99  1.93  3.48  3.00 |
| *N. subvirida* | Bio3: Isothermality  Bio7: Temperature annual range  Bio9: Mean temperature of driest quarter  Bio14: Precipitation of driest month  Bio15: Precipitation seasonality (coefficient of variation)  Bio16: Precipitation of wettest quarter | 1.57  1.71  1.84  2.05  2.84  1.52 |

## Generating a bias layer

To consider the bias in sampling effort a bias layer was generated by suggested methods by Rinnan (2015). This raster file was generated by the same resolution as other environmental variables at (1 x 1 km^2^) to consider the sampling intensity in the models using Maximum Entropy (*MaxEnt*) algorithm (Phillips et al. 2009; Fourcade et al. 2014). To generate the bias layer i) a raster file was generated form the occurrences (as the presence of the studied species) using *rasterize* function from *raster* package (V. 3.6-26; Hijmans et al. 2015), ii) we generated a two-dimensional kernel density layer as the estimation of occurrences density using kde2d function of MASS package (v. 7.3-60.0.1; Venables & Ripley 2013) and saved as a tif raster to be used in *MaxEnt* (Rinnan 2015).


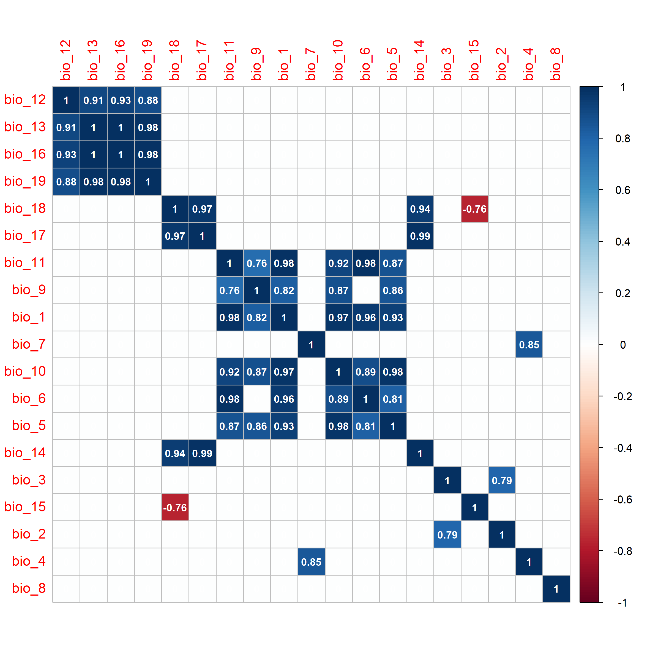

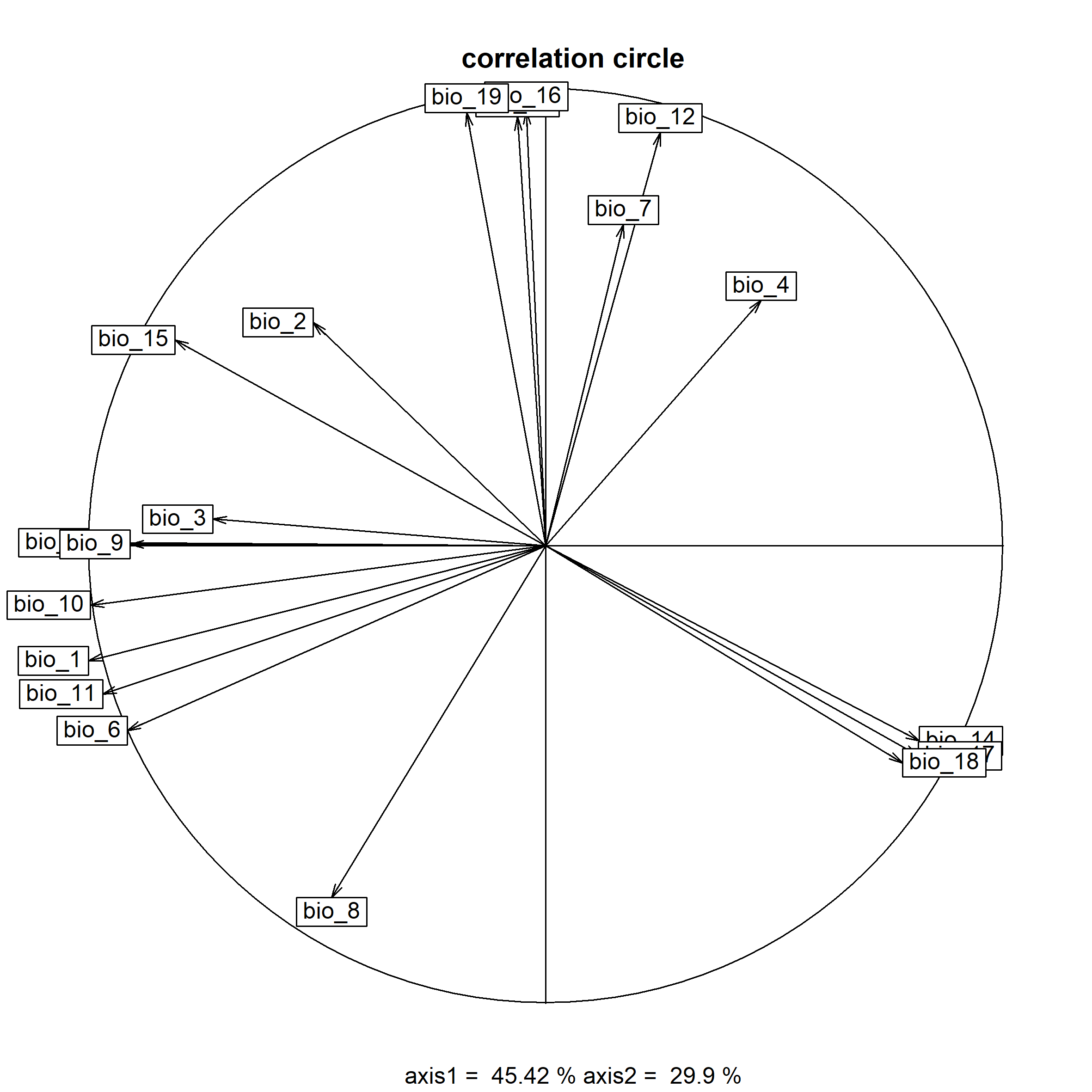

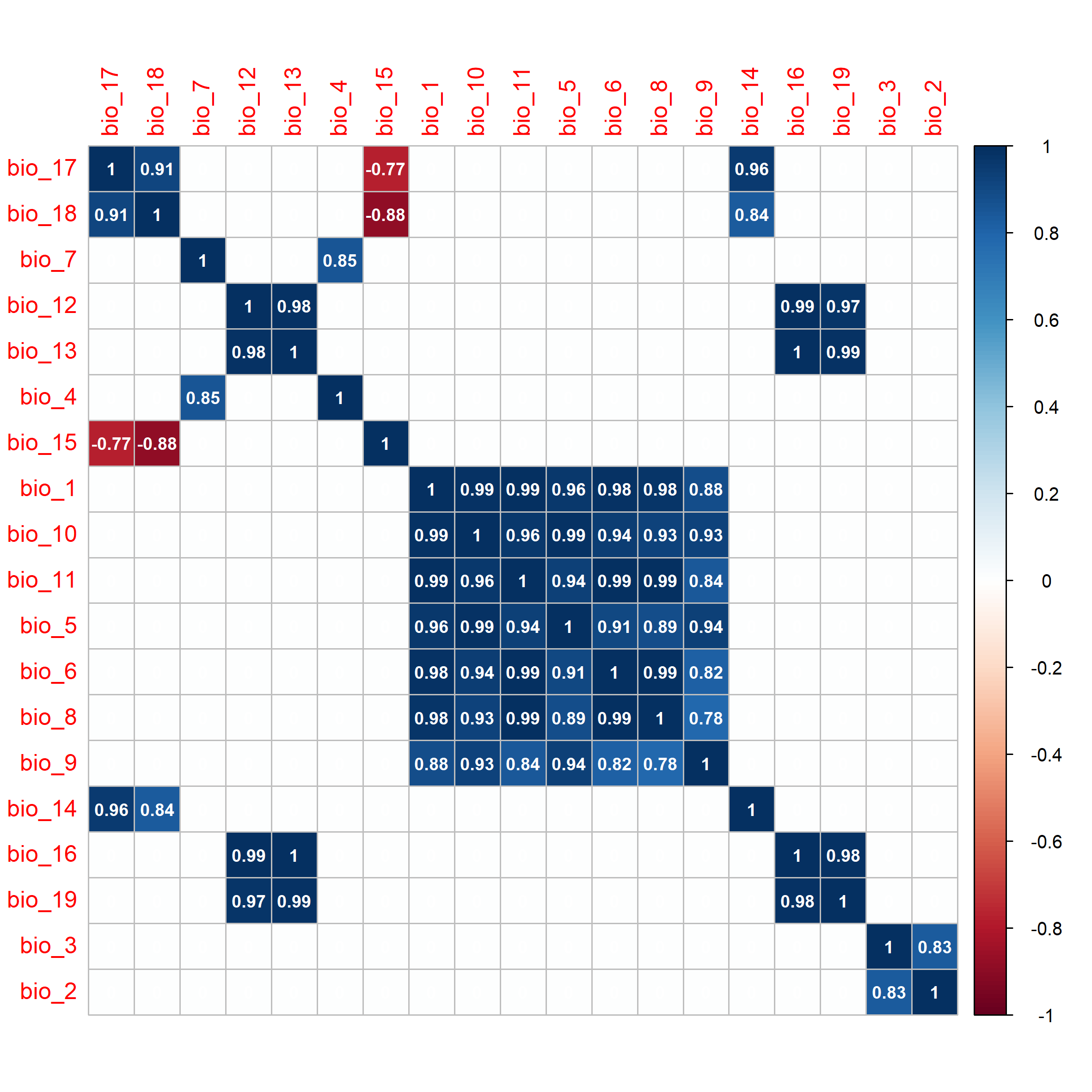

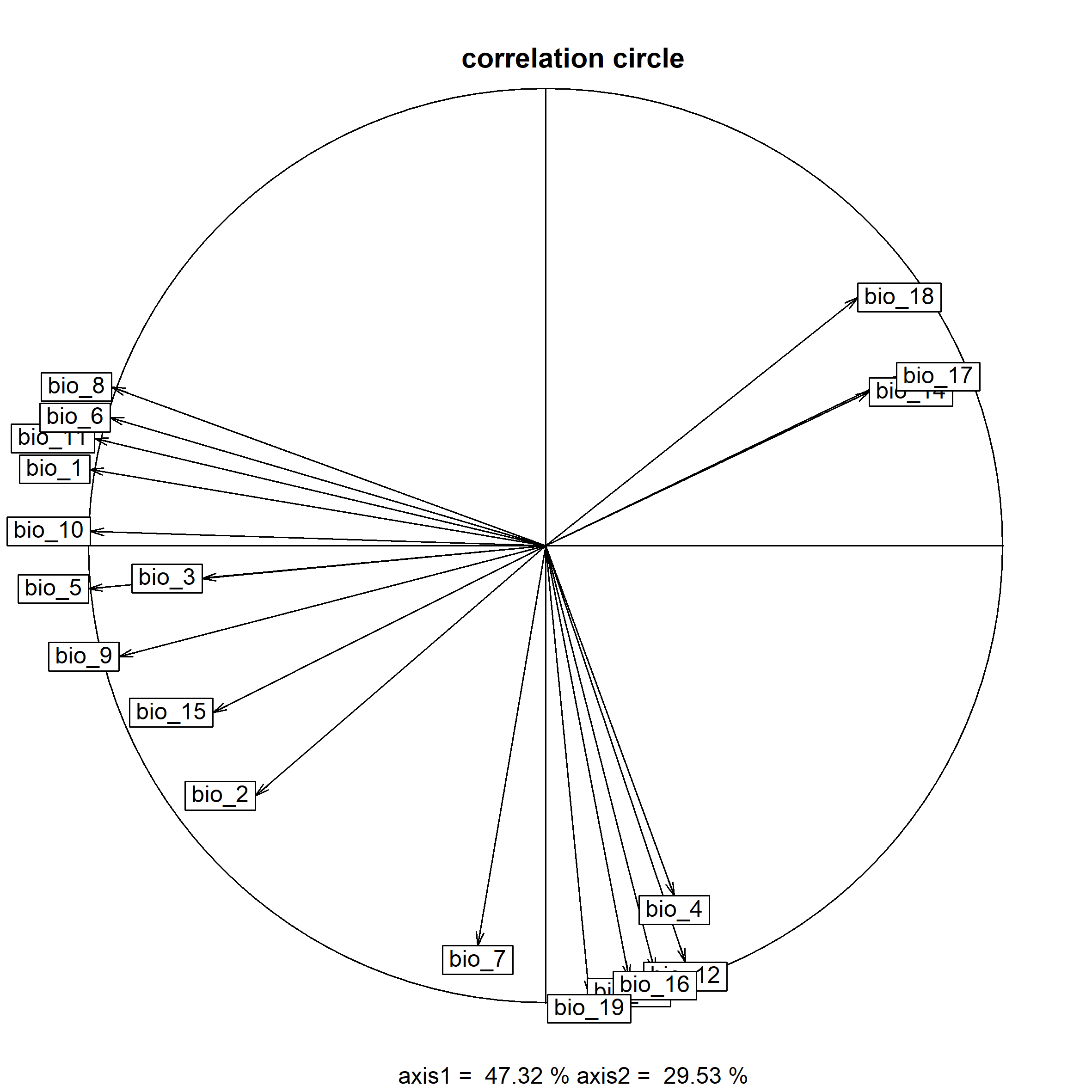


Figure S1. Results of pairwise Pearson's correlation coefficient (> 0.75 a and c) The Correlation circle of variables resulted from Principal Component Analysis (PCA) to select the most independent climate variables for the studied species (b and d). a and b) *N.divergaria*, and c and d) *N.subvirida* to test the multicollinearity problems.

**a**

**c**

**d**

**b**

Before fitting our models, first we cropped the selected environmental variables by the extent of a Minimum Convex Polygon (MCP), which was generated around the occurrences of species by a buffer of 100 km using the mcp function from *adehabitatHR* package in R programming language (Calenge 2006; R Core Team 2024). This extent covers all the climate space for the distribution of both species.

## MaxEnt tuning

In this study we used *MaxEnt* algorithm within *Biomod2* package to model the species distribution (Thuiller et al. 2024). 10,000 background/pseudo-absence points were randomly generated within the cropped climate space for each species. A robust model was generated using 10 replications of the models besides 80/20% split of the occurrences following suggested methodology by (Ginal et al. 2022 and Noori et al. 2024).

The most optimal models were selected to ensemble the final models based on values higher than 0.7 for AUC_test_ (Area under the ROC Curve) and TSS (True Skill Statistic) values to predict the areas with higher habitat suitability for the species (Table S1; full results are provided in Supplementary Materials 1). Furthermore, we set 10% of omission as threshold presence-absence across 100 replicates. Finally, we assessed the forecasted models by generating a layer for multivariate environmental similarity surfaces (MESS) Using mess function from *dismo* package (Elith et al. 2010). The assessed MESS was reclassified to 1 for those pixels that are within the training range and 0 for pixels that required extrapolations (Fig. S2 and S3; Rödder et al. 2013; Ginal et al. 2022).


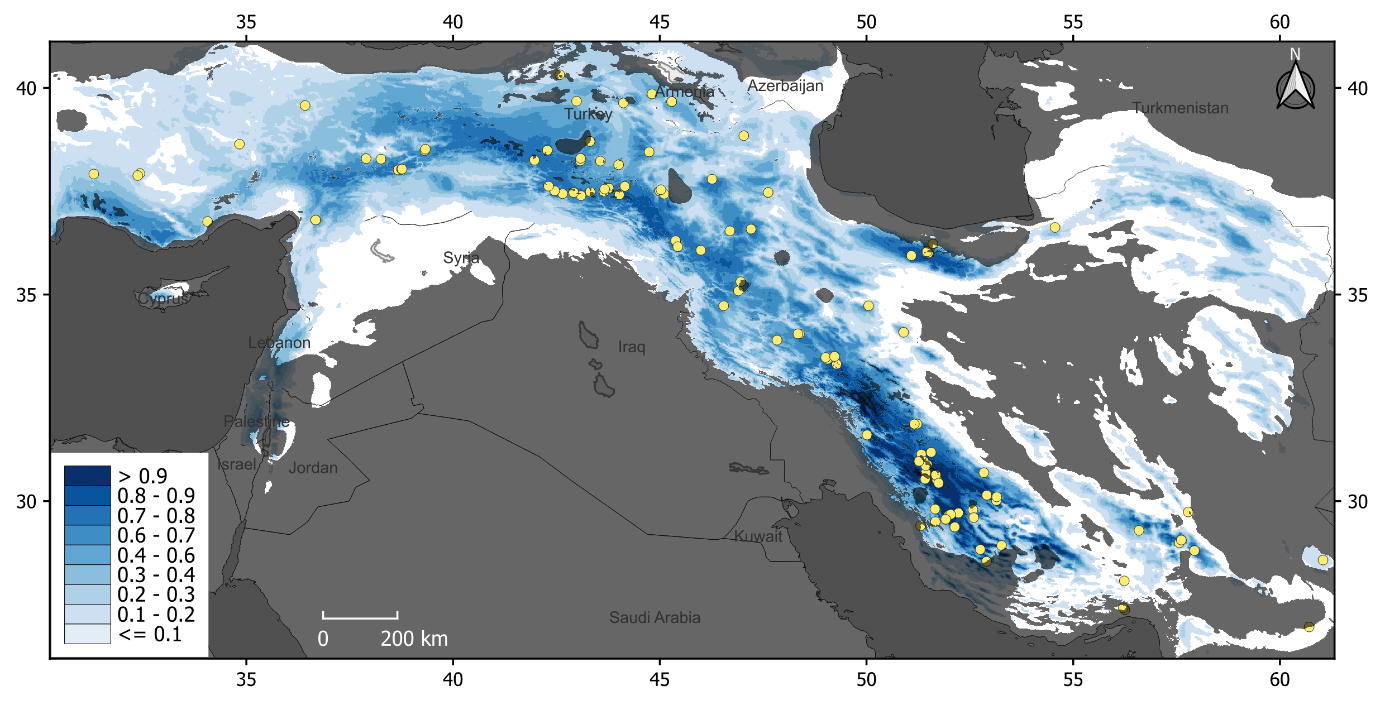


Figure S2. Ensembled model for distribution of the *N. divergaria*. Yellow points show the occurrences were used in SDMs. Higher values of the blue color display higher probability for habitat suitability. The dark color layer depicts MESS.


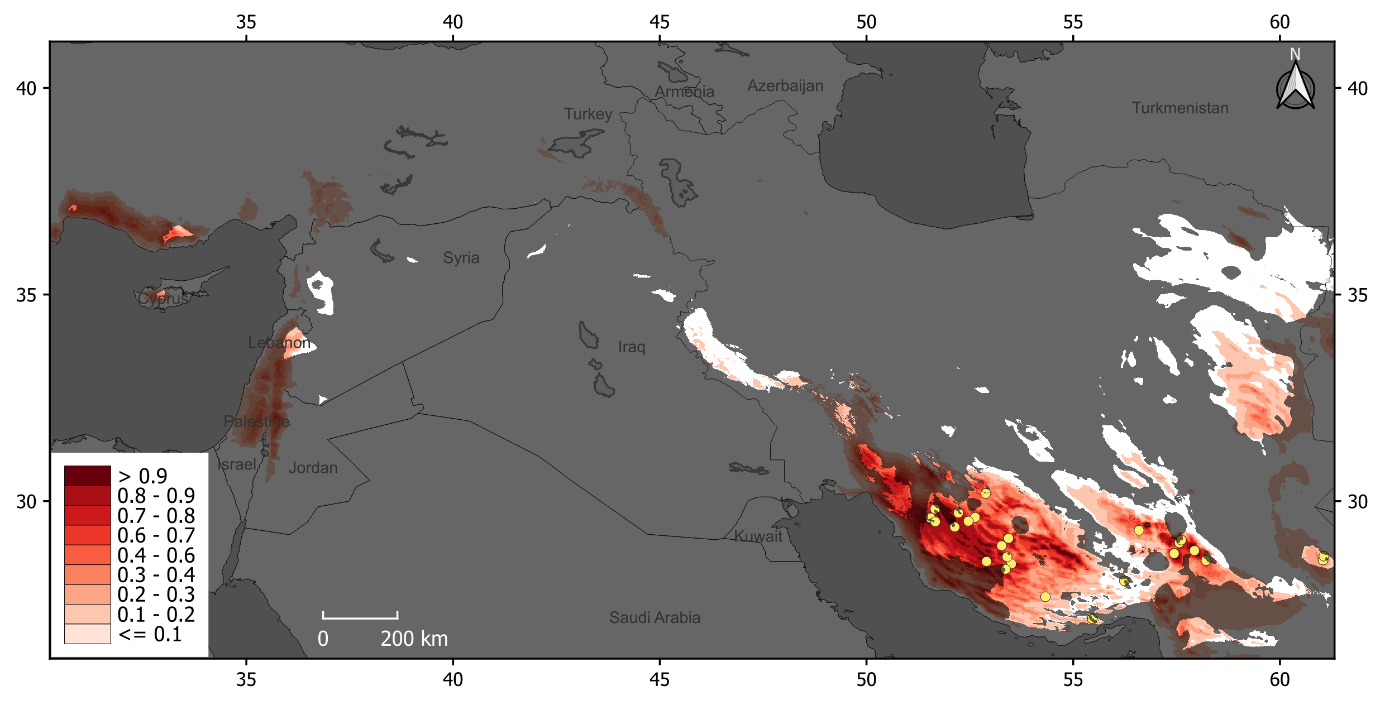


Figure S3. Ensembled model for distribution of the *N. subvirida*. Yellow points show the occurrences were used in SDMs. Higher values of the red color display higher probability for habitat suitability. The dark color layer depicts MESS.

**Climate space overlaps**

We used different metric to check for the extent of the climate space that has been occupied by two species of *Nychoides*. We used the resulting ensembled models by SDM for each species (species model; Fig. S2 and S3) to extract the values of the climate species for each species using R programming (R Core Team 2024). To do this, first we generated a Minimum Convex Polygon (MCP) with a buffer of 50 km around the occurrence data for each species using *rangeBuilder* package (Fig. 1; Davis Rabosky et al. 2016). Then the raster files of the species model were masked by this MCP (Fig. 1). We thus extracted the values of shared climate variables between two species (bio3, 7, 9, 14, and 15) for the sample of 500 points within each species’ cropped MCP. This climate variables were used to perform a PCA analysis using *prcomp* function. Consequently, we test the niche overlap between two species using niche qualification approach from *ecospat* package (Di Cola et al. 2017; Broennimann et al. 2024) using the first two PCA products. Resulting Schoener's D (D) and Hellinger’s I (I) assess the niche overlap between the studied species (Di Cola et al. 2017; Broennimann et al. 2024). These two metrics provide complementary measure for niche overlaps; while *D* considers the differences between habitat suitability of the different species, *I* indicates their similarity of habitat suitability probabilities (Table 3; Di Cola et al. 2017).

Furthermore, we plotted the PCA values (PC1 and PC2) for studied species to show the overlapping of the species’ niches across the accessible climate space for each species (Fig. S5-8). Additionally, we performed a Welch’s test (*ggstatsplot* package; Patil 2021) to see if the PCA values between two species are significantly different. We applied all these metrics for different threshold for habitat suitability (areas with probability more than 1%, 50%, 75%, and 85%; Fig. S4). This approach helps us to have a better understanding of niche overlaps of the studied species. As shows in Table 3, our niche qualification test show that the habitat preference for the studied species is significantly different from each other.


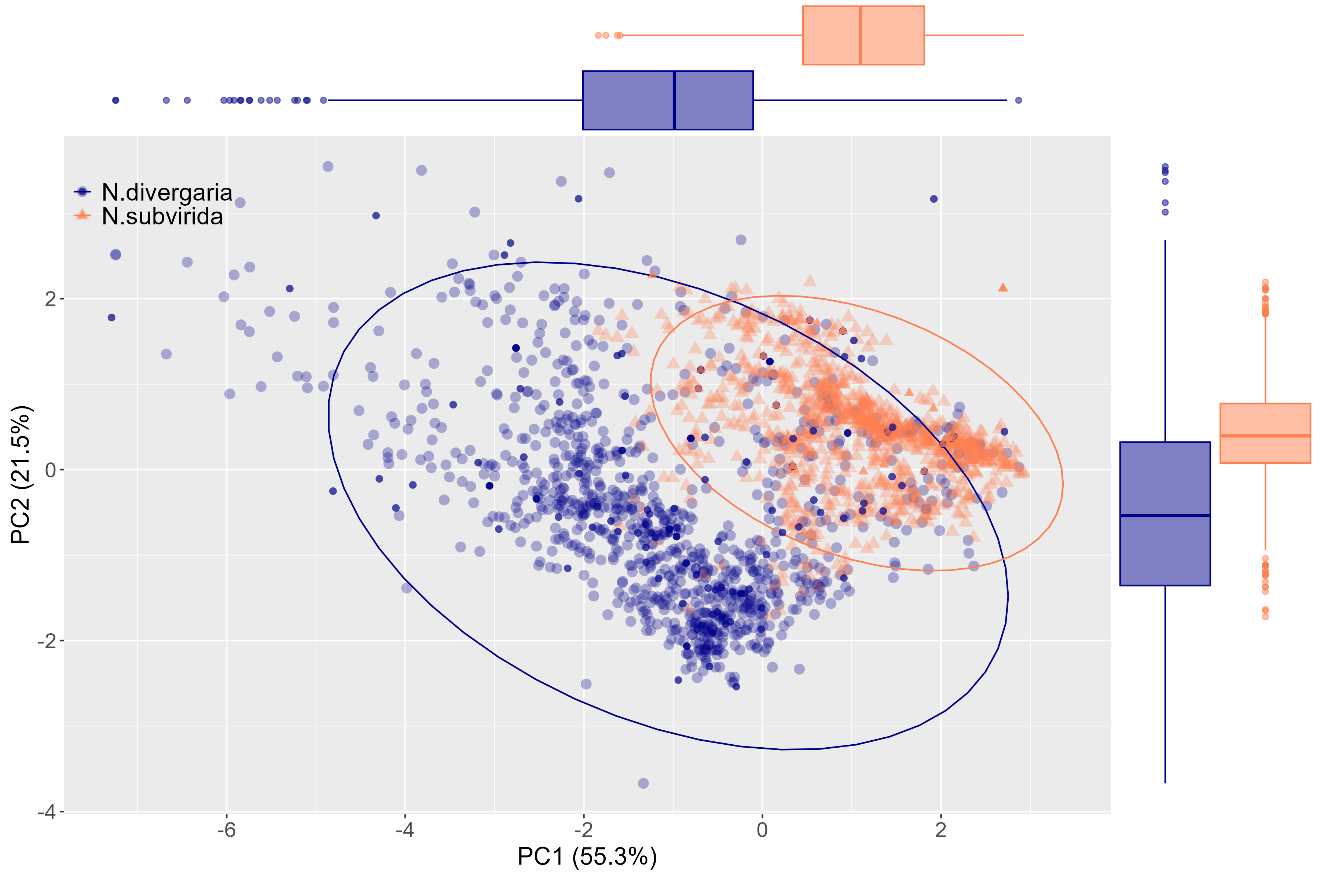


Figure S5. A comparison between occupied climate space by two *Nychoides species across the area with higher probability than 25% for the study area. The points within the ellipses show the 95% of the values for each species. the bigger shapes indicate the utilized occurrences in this study.*


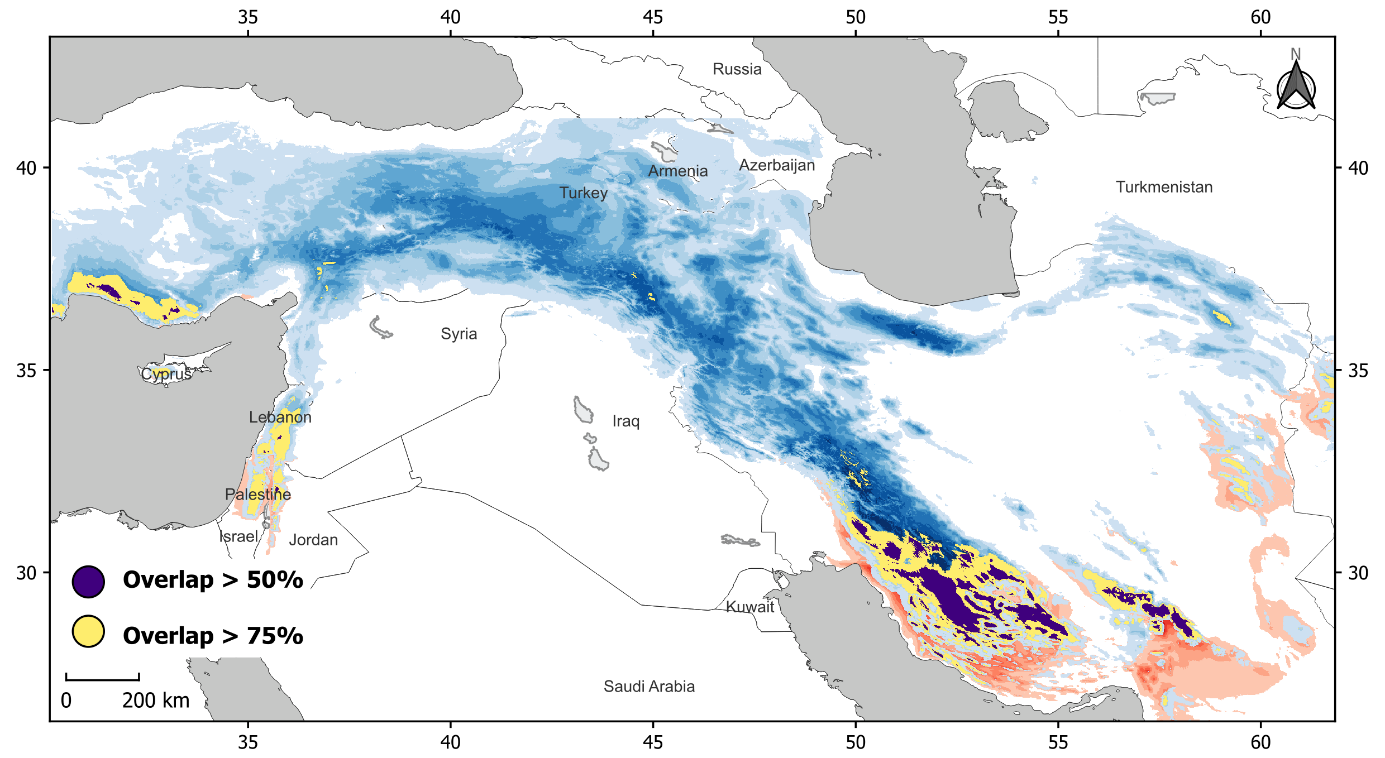


Figure S4. The overlapped areas with higher probability (> 25% and > 50%) habitat suitability layers between *N. divergaria and N. subvirida.*

**Overlap > 50%**

**Overlap > 25%**


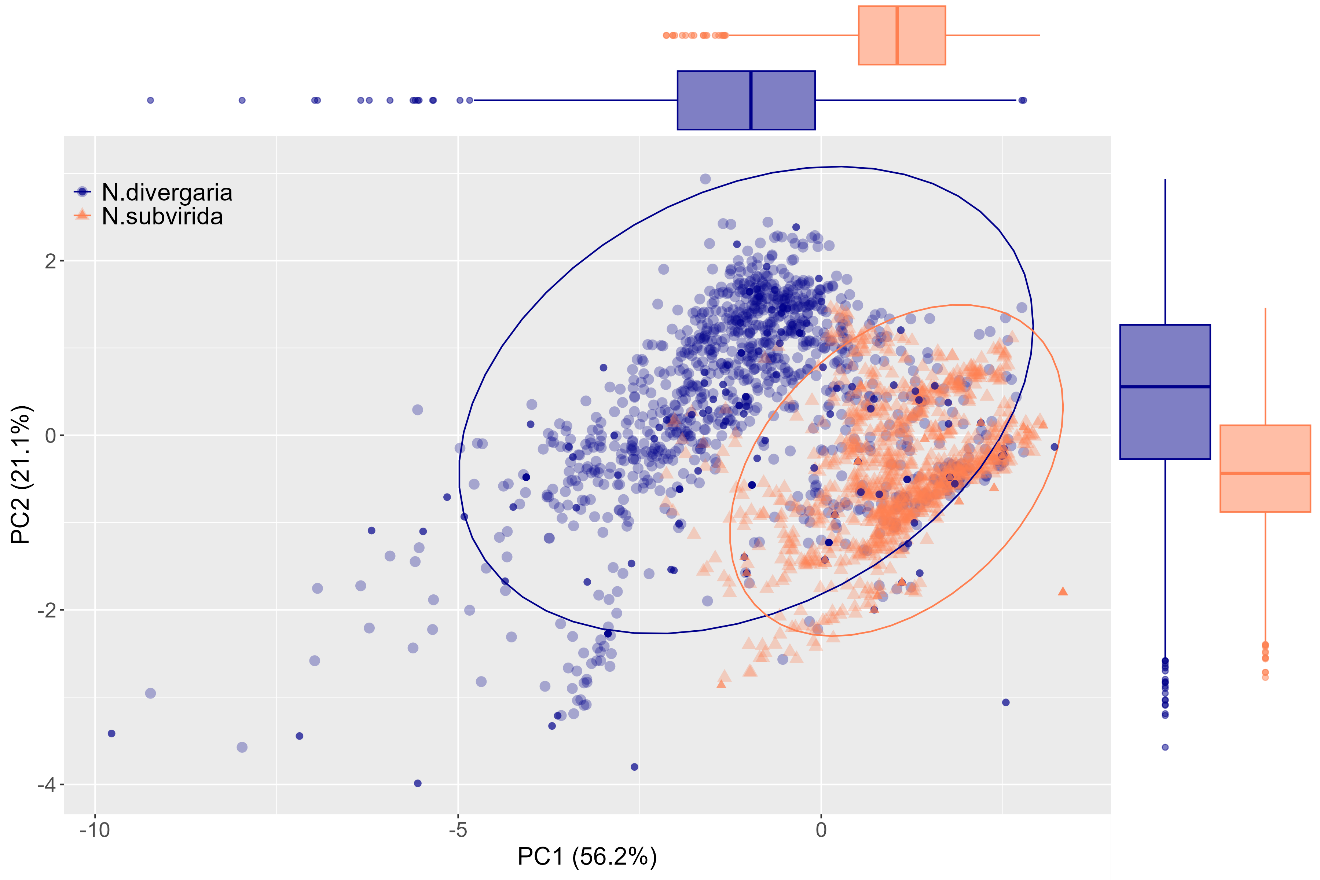


Figure S6. A comparison between occupied climate space by two *Nychoides species across the area with higher probability than 50% for the study area. The points within the ellipses show the 95% of the values for each species. the bigger shapes indicate the utilized occurrences in this study.*


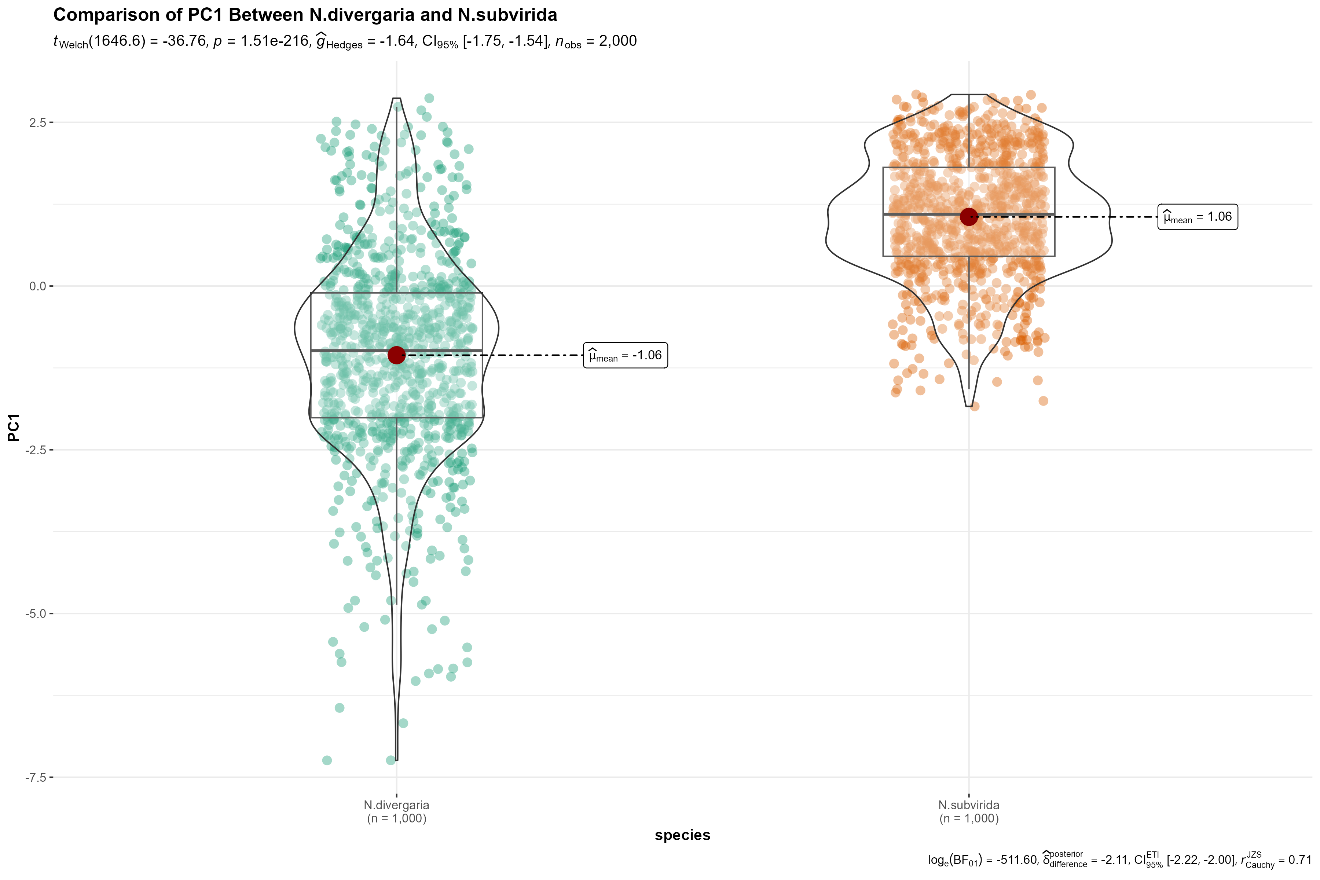


Figure S7. Comparison between PC1 values for two *Nychoides species across the area with higher probability than 25% for the study area.*


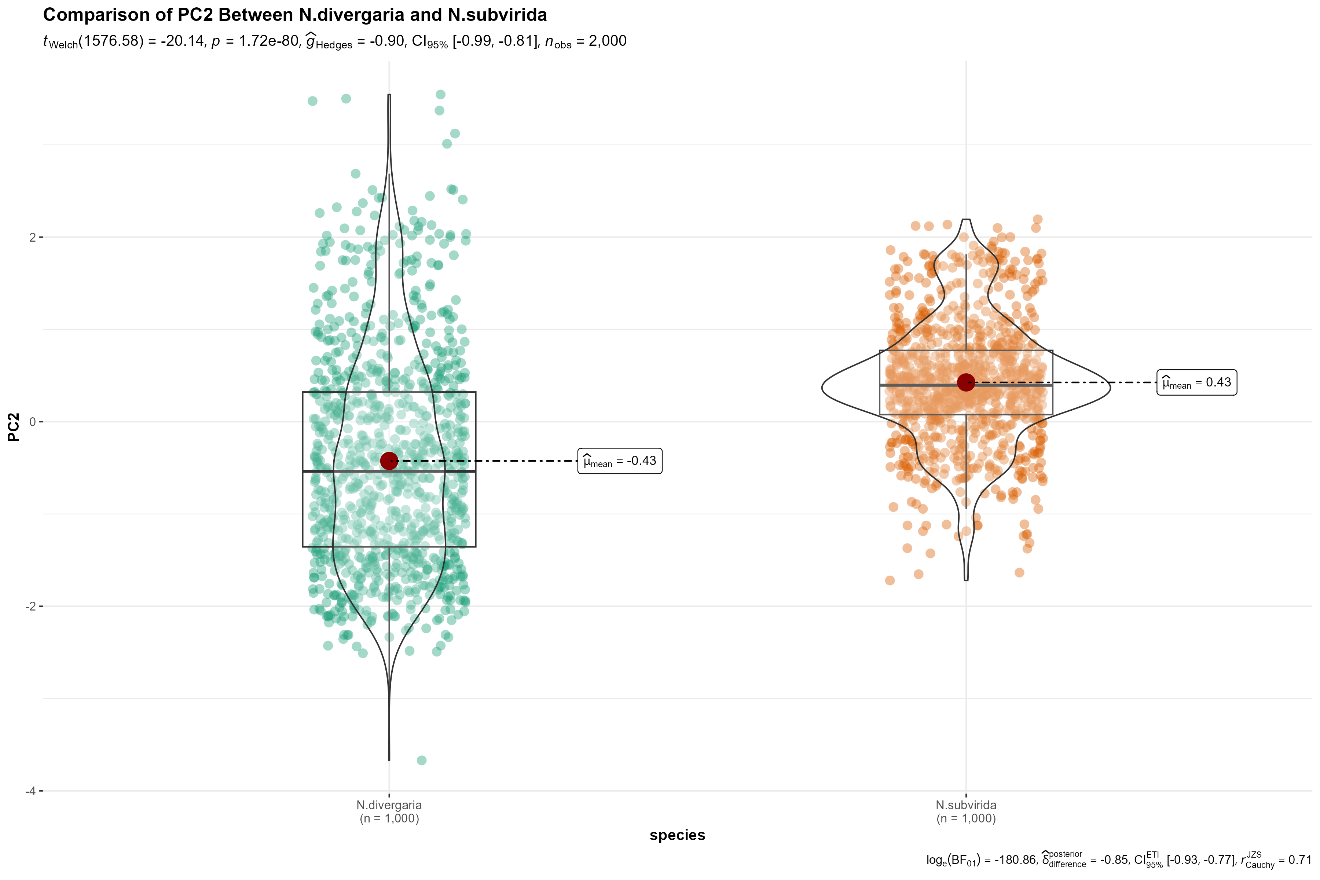


Figure S8. Comparison between PC2 values for two *Nychoides species across the area with higher probability than 25% for the study area.*


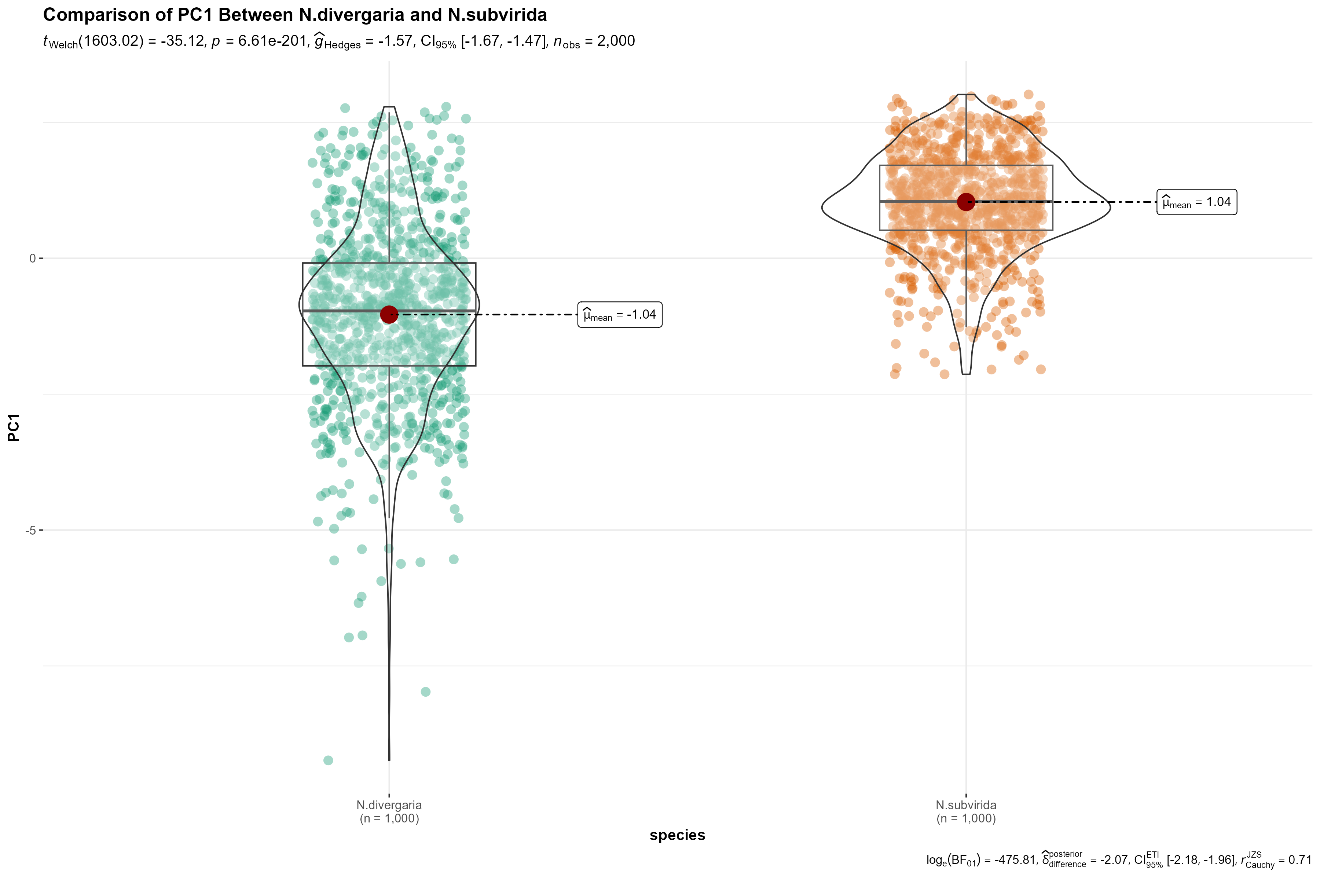


Figure S9. Comparison between PC1 values for two *Nychoides species across the area with higher probability than 50% for the study area.*


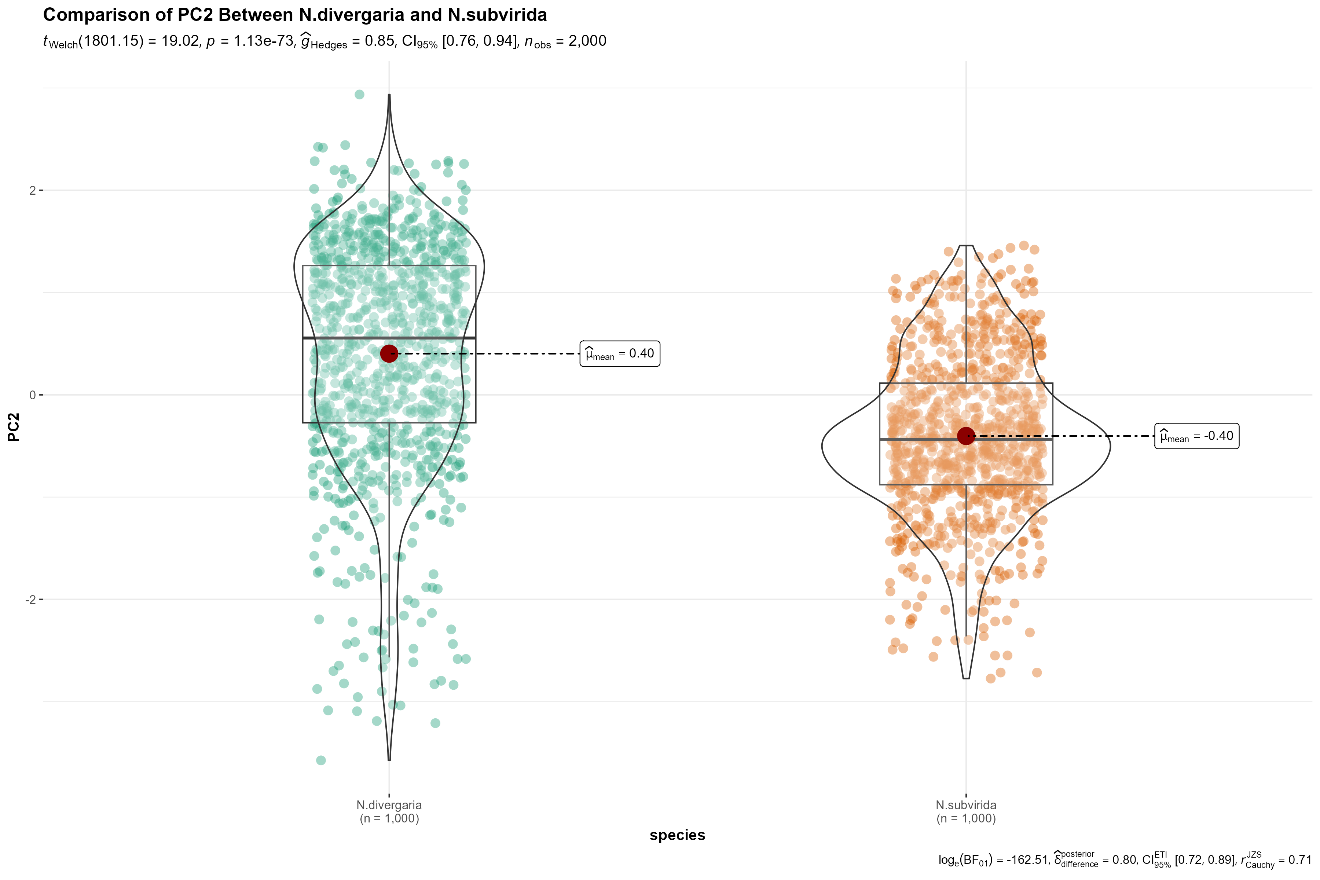


Figure S10. Comparison between PC2 values for two *Nychoides species across the area with higher probability than 50% for the study area.*


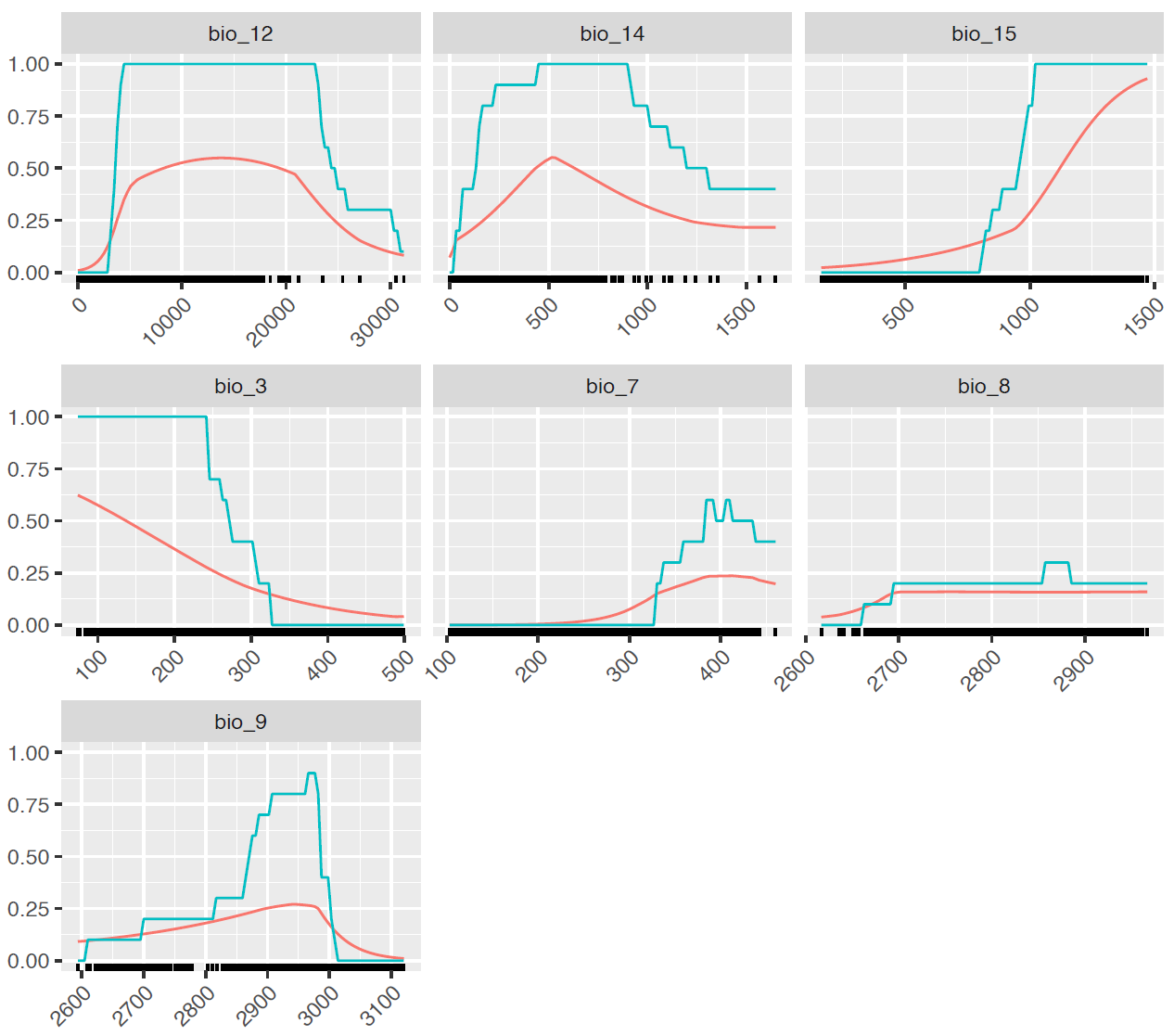


Figure S11. Response curves for climate variables contribute in SDMs of *N. divergaria*.


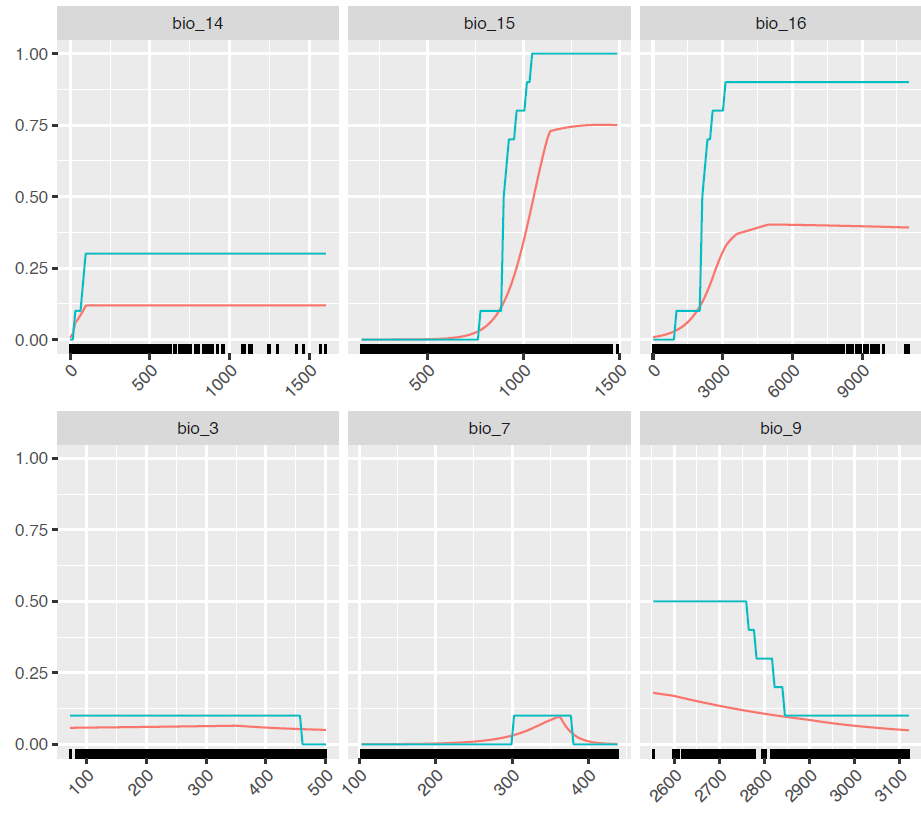


Figure S12. Response curves for climate variables contribute in SDMs of *N. subvirida*.

**Table S2.** Taxa used in this study with identification and GenBank accession numbers for each gene.

| Sample ID | Taxon | COI | ArgK | Ca-  ATPase | EF1a | RpS5 | wingless |
| --- | --- | --- | --- | --- | --- | --- | --- |
| SMNS-DNA-18 | *Nychiodes antiquaria* | OP629530 | - | - | OP721355 | OP721375 | OP721393 |
| SMNS-DNA-20 | *Nychiodes leviata* | OP629531 | OP721343 | OP721350 | OP721356 | OP721376 | OP721394 |
| SMNS-DNA-21 | *Nychiodes farinosa* | OP629532 | - | - | OP721357 | OP721377 | OP721395 |
| SMNS-DNA-23 | *Nychiodes subvirida* | OP629533 | OP721344 | - | OP721358 | OP721378 | - |
| SMNS-DNA-24 | *Nychiodes divergaria* | OP629534 | OP721345 | OP721351 | OP793636 | OP721379 | - |
| SMNS-DNA-25 | *Nychiodes divergaria* | OP629535 | - | - | OP721359 | OP721380 | - |
| SMNS-DNA-27 | *Nychiodes divergaria* | OP629536 | - | - | OP721360 | - | OP721396 |
| SMNS-DNA-28 | *Nychiodes subvirida* | OP629537 | - | - | OP721361 | OP721381 | - |
| SMNS-DNA-29 | *Nychiodes subvirida* | OP629538 | - | - | OP721362 | OP721382 | OP721397 |
| SMNS-DNA-31 | *Nychiodes divergaria* | OP629539 | - | - | OP721363 | - | OP721398 |
| SMNS-DNA-34 | *Nychiodes divergaria* | OP629540 | - | - | OP721364 | OP721383 | - |
| SMNS-DNA-35 | *Nychiodes divergaria* | OP629541 | OP721346 | OP721352 | OP793637 | OP721384 | OP721399 |
| SMNS-DNA-36 | *Nychiodes divergaria* | OP629542 | OP721347 | - | OP721365 | OP721385 | OP721400 |
| SMNS-DNA-39 | *Nychiodes divergaria* | OP629543 | - | OP721353 | OP721366 | OP721386 | OP721401 |
| SMNS-DNA-40 | *Nychiodes divergaria* | OP629544 | - | - | OP721367 | OP721387 | OP721402 |
| SMNS-DNA-41 | *Nychiodes divergaria* | OP629545 | OP721348 | OP721354 | OP721368 | OP721388 | OP721403 |
| SMNS-DNA-42 | *Nychiodes admirabila* | OP629546 | OP721349 | - | OP793638 | OP721389 | OP721404 |
| SMNS-DNA-49 | *Nychiodes divergaria* | OP629547 | - | - | OP721369 | - | OP721405 |
| SMNS-DNA-53 | *Nychiodes divergaria* | OP629548 | - | - | OP721370 | - | OP721406 |
| SMNS-DNA-54 | *Nychiodes divergaria* | OP629549 | - | - | OP793639 | - | OP721407 |
| SMNS-DNA-58 | *Nychiodes subvirida* | OP629550 | - | - | OP721371 | - | OP721408 |
| SMNS-DNA-62 | *Nychiodes divergaria* | - | - | - | OP721372 | OP721390 | OP721409 |
| SMNS-DNA-63 | *Nychiodes divergaria* | OP629551 | - | - | OP721373 | OP721391 | OP721410 |
| SMNS-DNA-89 | *Nychiodes divergaria* | OP629552 | - | - | OP721374 | OP721392 | - |

**References**

Calenge C (2006) The package “adehabitat” for the R software: a tool for the analysis of space and habitat use by animals. Ecological modelling 197 (3–4): 516–519.

<https://doi.org/10.1016/j.ecolmodel.2006.03.017>

Davis Rabosky AR, Cox CL, Rabosky DL, Title PO, Holmes IA, Feldman A, McGuire JA (2016) Coral snakes predict the evolution of mimicry across New World snakes. Nature communications 7(1): 11484.

<https://doi.org/10.1038/ncomms11484>

Di Cola V, Broennimann O, Petitpierre B, Breiner FT, D'Amen M, Randin C, Engler R, Pottier J, Pio D, Dubuis A, Pellissier L, Mateo RG, Hordijk W, Salamin N, Guisan A (2017) ecospat: an R package to support spatial analyses and modeling of species niches and distributions. Ecography 40(6): 774–787.

<https://doi.org/10.1111/ecog.02671>

Dray S, Dufour A (2007) The ade4 Package: Implementing the Duality Diagram for Ecologists. Journal of Statistical Software 22(4): 1–20.

<https://doi.org/10.18637/jss.v022.i04>

Fourcade Y, Engler JO, Rödder D, Secondi J (2014) Mapping species distributions with MAXENT using a geographically biased sample of presence data: a performance assessment of methods for correcting sampling bias. PloS one 9(5): e97122.

<https://doi.org/10.1371/journal.pone.0097122>

Hijmans RJ, Van Etten J, Cheng J, Mattiuzzi M, Sumner M, Greenberg JA, Hijmans MRJ (2015) Package ‘raster’. R package, 734, 473.

Ginal P, Tan WC, Rödder D (2022) Invasive risk assessment and expansion of the realized niche of the Oriental Garden Lizard *Calotes versicolor* species complex (Daudin, 1802). Frontiers of Biogeography 14(3).

<https://doi.org/10.21425/F5FBG54299>

Karger DN, Conrad O, Böhner J, Kawohl T, Kreft H, Soria-Auza RW, Zimmermann NE, Linder HP, Kessler M (2017) Climatologies at high resolution for the earth’s land surface areas. Scientific data 4(1): 1–20.

<https://doi.org/10.1038/sdata.2017.122>

Noori S, Hofmann A, Rödder D, Husemann M, Rajaei H (2024a) A window to the future: effects of climate change on the distribution patterns of Iranian Zygaenidae and their host plants. Biodiversity and Conservation 33(2): 579–602.

<https://doi.org/10.1007/s10531-023-02760-2>

Patil I (2021) Visualizations with statistical details: The 'ggstatsplot' approach. Journal of Open Source Software 6(61): 3167.

<https://doi.org/10.21105/joss.03167>

Phillips SJ, Dudík M, Elith J, Graham CH, Lehmann A, Leathwick J, Ferrier S (2009) Sample selection bias and presence‐only distribution models: implications for background and pseudo‐absence data. Ecological applications 19(1). 181–197.

<https://doi.org/10.1890/07-2153.1>

R Core Team (2024) R: A Language and Environment for Statistical Computing. R Foundation for Statistical Computing, Vienna, Austria. URL: <https://www.R-project.org/>.

Rinnan DS (2015) How to construct a bias file with R for use in MaxEnt modeling. Retrieved from: <https://scottrinnan.wordpress.com/>

Rödder D, Lawing AM, Flecks M, Ahmadzadeh F, Dambach J, Engler JO, Habel JC, Hartmann T, Hörnes D, Ihlow F, Schidelko K, Stiels D, Polly PD (2013) Evaluating the significance of paleophylogeographic species distribution models in reconstructing Quaternary range-shifts of Nearctic chelonians. PLoS One 8(10): e72855.

<https://doi.org/10.1371/journal.pone.0072855>

Thuiller W, Georges D, Gueguen M, Engler R, Breiner F, Lafourcade B, Patin R, Blancheteau H (2024) biomod2: Ensemble Platform for Species Distribution Modeling. R package version 4.2-5-2.

<https://CRAN.R-project.org/package=biomod2>.

Venables WN, Ripley BD (2013) Modern applied statistics with S-PLUS third edition. Springer Science.
